# Functional saturation of Earth’s habitable domains: constraints on land systems and potential in marine environments

**DOI:** 10.3389/fsysb.2026.1941138

**Published:** 2026-09-16

**Authors:** I. Valentin Petrescu-Mag, Ioan G. Oroian, Benone Păsărin, O. Călin Safirescu, Petru Burduhos, Ioan Braşovean, Daniela Bordea, Bianca Moldovan, Tudor N. Pojar, Ioan Bănățean-Dunea, F. Camelia Oroian, Praveen Pandey

**Affiliations:** 1 Department of Environmental Engineering and Protection, Faculty of Agriculture, University of Agricultural Sciences and Veterinary Medicine, Cluj-Napoca, Romania; 2 Doctoral School of Engineering Science, University of Oradea, Oradea, Romania; 3 Bioflux SRL, Cluj-Napoca, Romania; 4 Balkan Environmental Association (BENA), Thessaloniki, Greece; 5 Department of Management of Animal Resources and Technologies, Faculty of Food and Animal Sciences, “Ion Ionescu de la Brad” University of Life Sciences, Iaşi, Romania; 6 EBBSA International, Niš, Serbia; 7 Department of Animal Breeding and Animal Production, Faculty of Veterinary Medicine, University of Agricultural Science and Veterinary Medicine, Cluj-Napoca, Romania; 8 European Federation of Animal Science, European Association for Animal Production (EAAP), Rome, Italy; 9 Biology and Plant Protection Department, Faculty of Agriculture, University of Life Sciences “King Mihai I” from Timişoara, Timişoara, Romania; 10 Department of Economic Sciences, Faculty of Horticulture and Business for Rural Development, University of Agricultural Sciences and Veterinary Medicine, Cluj-Napoca, Romania; 11 Division of Plant Breeding and Genetic Resource Conservation, CSIR - Central Institute of Medicinal and Aromatic Plants, Lucknow, India

**Keywords:** anthropogenic spatial saturation, biosphere resilience, ecological asymmetry, land-system transformation, macroalgal productivity dynamics, ocean acidification feedbacks, regime shift vulnerability

## Abstract

In this context, functional saturation is defined as the state in which a system’s capacity to sustain additional productive or regulatory functions becomes constrained due to cumulative anthropogenic pressures, even if some physical space remains available. This review examines whether the progressive contraction of terrestrial plant communities under intensifying anthropogenic pressure can be functionally compensated by the expansion of marine macroalgal systems in a high-CO_2_ world. Integrating perspectives from Earth system ecology, biogeochemistry, and spatial analysis, the study evaluates three interdependent dimensions: the saturation of habitable terrestrial space, the relative openness yet ecological constraint of marine environments, and the dynamic coupling between atmospheric CO_2_ and ocean chemistry. The synthesis reveals a fundamental asymmetry between land and ocean systems. Terrestrial ecosystems are undergoing direct structural transformation through land-use change, resulting in a measurable contraction of ecologically functional space and increasing transgression of planetary boundaries. In contrast, marine systems retain substantial geometric space but are subject to diffuse, cumulative stressors, particularly in productive coastal zones where macroalgal communities are most viable. Elevated CO_2_ exerts dual effects on marine primary producers, simultaneously enhancing photosynthetic potential while intensifying ocean acidification, with temporally lagged responses mediated by air–sea exchange dynamics. Although macroalgae may exhibit localized increases in productivity, their capacity to provide long-term carbon sequestration and ecosystem stability remains inherently limited due to rapid biomass turnover, susceptibility to regime shifts, and the interaction of multiple stressors. The available evidence suggests that marine macroalgal expansion is unlikely to fully compensate for terrestrial vegetation loss at global ecological and biogeochemical scales. Instead, the Earth system is undergoing a net reduction in biospheric resilience, characterized by declining terrestrial complexity and increasingly unstable marine productivity. These findings challenge assumptions of compensatory redistribution of primary production and underscore the necessity of preserving terrestrial ecosystems alongside mitigating pressures on marine environments.

## 1 Introduction

Angiosperms are conventionally regarded as the most successful lineage of terrestrial plants, representing the culmination of a long evolutionary trajectory characterized by increasing structural complexity, ecological versatility, and reproductive efficiency (Zuntini et al., 2024; Berger et al., 2023; Friis et al., 2025; Benton et al., 2021). Their extensive diversification, particularly during the Cretaceous and early Cenozoic, unfolded within a specific geological and atmospheric context that facilitated the expansion of terrestrial ecosystems and the stabilization of plant–environment interactions (Berger et al., 2023; Friis et al., 2025; Ramírez-Barahona et al., 2020; Benton et al., 2021). Implicit in this perspective is the assumption that angiosperms, once established as dominant primary producers on land, will continue to occupy this position under future planetary conditions (Berger et al., 2023; Ramírez-Barahona et al., 2020; Benton et al., 2021).

However, the present state of the Earth system raises legitimate questions regarding the persistence of this dominance (Sage et al., 2024; Ding et al., 2025; Zhao and Running, 2010; Hou et al., 2022). Contemporary global change—encompassing rising atmospheric carbon dioxide concentrations, large-scale anthropogenic disturbance, and increasing pressure on available physical space—introduces a set of constraints and drivers that differ fundamentally from those under which angiosperms achieved their evolutionary success (Sage et al., 2024; Ding et al., 2025; Zhao and Running, 2010; Hou et al., 2022) (Table 1). These transformations do not merely affect the performance of individual taxa but may alter the relative balance between major groups of primary producers (Sage et al., 2024; Ding et al., 2025; Kwiatkowski et al., 2020; Bopp et al., 2013).

**TABLE 1:** Contrasting terrestrial vs. marine responses to CO_2_ and climate.

Factor	Terrestrial angiosperms	Marine macroalgae	References
CO_2_ rise and climate	Alters NPP, drought impacts, land-use interactions	Alters productivity via acidification, warming, light	Shamir et al. (2021), Sage et al. (2024), Gao et al. (2012), Ding et al. (2025), Kwiatkowski et al. (2020), Pessarrodona et al. (2022), Gao et al. (2021), Zhao and Running (2010), Hou et al. (2022), Bopp et al. (2013)

NPP, Net Primary Productivity.

Within this framework, three interrelated factors merit particular consideration: (1) the proximity and intensity of anthropogenic influence on terrestrial environments, (2) the ongoing increase in atmospheric and oceanic carbon dioxide, and (3) the finite and progressively saturated nature of habitable land space (Sage et al., 2024; Ding et al., 2025; Zhao and Running, 2010; Hou et al., 2022). Each of these factors has the potential to reshape ecological competitiveness, resource availability, and spatial distribution at a global scale (Sage et al., 2024; Ding et al., 2025; Kwiatkowski et al., 2020; Zhao and Running, 2010; Hou et al., 2022; Bopp et al., 2013).

In contrast to terrestrial vascular plants, marine macroalgae (commonly referred to as seaweeds) occupy an environment that differs markedly in its exposure to these constraints (Shamir et al., 2021; Gao et al., 2012; Kwiatkowski et al., 2020; Pessarrodona et al., 2022; Gao et al., 2021; Nagelkerken and Connell, 2015; Fujita et al., 2023). The marine realm, while not exempt from human impact, offers vast spatial continuity, distinct carbon dynamics, and, in certain contexts, reduced direct exploitation compared to terrestrial systems (Shamir et al., 2021; Gao et al., 2012; Kwiatkowski et al., 2020; Pessarrodona et al., 2022; Gao et al., 2021; Nagelkerken and Connell, 2015; Fujita et al., 2023). This raises a provocative but as yet unresolved question: under the combined pressures of global change, could the balance of primary productivity shift in favor of marine macroalgae, allowing them to expand their ecological role beyond current limits (Shamir et al., 2021; Gao et al., 2012; Kwiatkowski et al., 2020; Pessarrodona et al., 2022; Gao et al., 2021; Nagelkerken and Connell, 2015; Fujita et al., 2023; Bopp et al., 2013) and potentially challenge the long-standing dominance of angiosperms?

The present work does not seek to provide an immediate answer to this question. Rather, it aims to explore the conditions under which such a shift might become conceivable, by examining the interaction between anthropogenic pressure, carbon dynamics, and spatial constraints, and by situating both angiosperms and marine macroalgae within this evolving environmental context (Shamir et al., 2021; Sage et al., 2024; Gao et al., 2012; Ding et al., 2025; Kwiatkowski et al., 2020; Pessarrodona et al., 2022; Gao et al., 2021; Zhao and Running, 2010; Nagelkerken and Connell, 2015; Fujita et al., 2023; Benton et al., 2021; Hou et al., 2022; Bopp et al., 2013).

## 2 Literature selection methodology

The literature survey underlying this study was conducted through a structured search strategy designed to capture the interdisciplinary nature of Earth-system processes, with a particular focus on the asymmetry between terrestrial and marine domains. Searches were performed across major scientific databases, including Google Scholar, CABI, Scopus, and Web of Science, ensuring broad coverage of peer-reviewed literature spanning ecology, biogeochemistry, global change biology, and oceanography.

Search queries were constructed using combinations of domain-specific keywords and Boolean operators to reflect the central themes of the study. Core keyword clusters included: terrestrial ecosystems, marine ecosystems, macroalgae, land-use change, ocean CO_2_ uptake, atmospheric CO_2_, primary productivity, planetary boundaries, ecosystem saturation, and anthropogenic impacts. These were combined using logical operators (AND, OR) to refine results and ensure both specificity and inclusiveness of relevant studies.

Inclusion criteria prioritized recent, high-impact, and methodologically robust studies, particularly those providing global syntheses, long-term datasets, or comparative analyses across environmental domains. Special emphasis was placed on literature addressing (i) spatial and functional constraints in terrestrial systems, (ii) biogeochemical dynamics of ocean carbon uptake, and (iii) the ecological role and productivity of marine macroalgal assemblages under changing environmental conditions. Foundational and widely cited earlier works were also included where necessary to provide conceptual grounding.

Exclusion criteria involved studies with narrow geographic scope lacking broader applicability, insufficient methodological transparency, or limited relevance to the comparative framework of terrestrial versus marine systems. Where appropriate, gray literature and reports were considered selectively to complement peer-reviewed findings, particularly in areas related to global assessments and environmental thresholds.

The final corpus was synthesized qualitatively, with emphasis on identifying convergent patterns, systemic differences, and emergent properties across domains. Rather than a purely quantitative meta-analysis, the approach followed an integrative synthesis model, suitable for capturing complex interactions among land-use dynamics, ocean chemistry, and biospheric responses under anthropogenic pressure.

## 3 Anthropogenic impacts on terrestrial plants vs. marine macroalgae

Human activities now shape most terrestrial landscapes and coastal seas, altering biodiversity patterns, ecosystem functioning and the persistence of plant and macroalgal communities (Sujatmiko et al., 2025; Sahavacharin et al., 2022; Muksin et al., 2020; Petrescu-Mag et al., 2024). Terrestrial vegetation generally experiences more direct, continuous and spatially extensive human pressure (Pendrill et al., 2022; Petrescu et al., 2020), whereas marine macroalgae are strongly impacted by climate-driven extremes and local coastal stressors (Sujatmiko et al., 2025; Pandey et al., 2026).

### 3.1 Spatial proximity and intensity of anthropogenic pressure on land

Global human modification and Human Footprint datasets show that about 75% of the planet’s land surface experiences measurable human pressure, with only roughly 43% of terrestrial lands in ∼2022 remaining at very low modification, and 10% already highly modified (Theobald et al., 2025; Venter et al., 2016). Between 1990 and 2020, 24% of terrestrial ecosystems (31 million km^2^) increased in modification (Theobald et al., 2025). From 2000 to 2018, more than 2 million km^2^ of wilderness (Human Footprint <1) were lost; mangrove dominated biomes suffered >5% wilderness loss, illustrating strong coastal pressure zones (Mu et al., 2022).

These pressures translate into substantial local biodiversity impacts in terrestrial plant assemblages. Globally, land use conversion and degradation have already reduced local terrestrial species richness by 13.6% on average and total abundance by 10.7%, with worst affected habitats showing 76.5% declines in local richness and 39.5% declines in abundance (Newbold et al., 2015). Land/sea use change emerges as the dominant direct driver of biodiversity loss, with direct exploitation second and pollution third; climate change and invasive species are comparatively less important on land than habitat conversion and exploitation (Jaureguiberry et al., 2022). Across realms, human pressures decrease local diversity and shift community composition, though without a clear global pattern of homogenization (Keck et al., 2025). When “community completeness” is considered, highly impacted regions retain on average < 20% of their regionally suitable plant species locally, compared with about 35% in areas with minimal human footprint (Pärtel et al., 2025).

Human consumption patterns further externalize terrestrial biodiversity loss: food consumption alone accounts for about 40% of global biodiversity loss, and more than half of the biodiversity loss driven by consumption in developed economies occurs outside their borders (Wilting et al., 2017). Overall, the terrestrial signal is one of pervasive, spatially extensive, and often chronic proximity between plants and intense anthropogenic modification (Table 2).

**TABLE 2:** Key quantitative indicators of terrestrial human pressure and plant biodiversity loss.

Indicator/effect (terrestrial)	Approximate magnitude	References
Land surface with measurable human pressure	∼75% of land	Venter et al. (2016)
Land with very low/low/moderate/high modification	43%/27%/20%/10% of land (2022)	Theobald et al. (2025)
Terrestrial ecosystems with increased modification (1990–20)	24% (31 million km^2^)	Theobald et al. (2025)
Wilderness loss 2000–2018 (human footprint <1)	>2 million km^2^; >5% loss in mangrove biomes	Mu et al. (2022)
Global average change in local species richness	−13.6%	Newbold et al. (2015)
Worst-affected habitats: richness/abundance change	−76.5% richness; −39.5% abundance	Newbold et al. (2015)
Community completeness in low vs. high footprint regions	∼35% vs. <20% of suitable species present locally	Pärtel et al. (2025)

### 3.2 Comparative drivers: terrestrial plants vs. marine assemblages and macroalgae

A global synthesis across realms shows that human pressures consistently reduce local diversity and shift community composition in terrestrial, freshwater and marine ecosystems, but the driver hierarchy and strength differ (Keck et al., 2025; Jaureguiberry et al., 2022). On land, land use change overwhelmingly dominates, while in the oceans direct exploitation and climate change are the primary drivers (Jaureguiberry et al., 2022). Marine biodiversity change at local scales is characterized by rapid compositional reorganization (Petrescu-Mag, 2026), with marine biomes often showing stronger and more variable changes than terrestrial ones; assemblage richness on average does not consistently decline, but some marine locations experience richness changes of up to about ±20% per year (Blowes et al., 2019).

Macroalgae (seaweeds) are major marine primary producers and structural habitat formers (Hanley et al., 2023; Fulton et al., 2019; Torres et al., 2025). Anthropogenic stressors affecting them include ocean warming and marine heatwaves, overfishing of herbivore predators, eutrophication, pollution, and marine litter (Hanley et al., 2023; Wernberg et al., 2023; El Qoraychy et al., 2023). Climate change–related warming and heatwaves stand out as pervasive threats for marine foundation species including kelps, often acting synergistically with other stressors such as overfishing and coastal development (Wernberg et al., 2023). In contrast to terrestrial plants, habitat loss *per se* is noted to be “much less keenly felt in the oceans than on land” for macroalgal systems (Hanley et al., 2023). Instead, climate driven extremes, trophic imbalances, and localized pollution events strongly shape macroalgal trajectories.

For instance, bull kelp forests in northern California lost >90% of canopy along more than 350 km of coastline following a marine heatwave and sea star mass mortality, leading to persistent urchin barrens and ∼80% abalone mortality (Rogers-Bennett and Catton, 2019; McPherson et al., 2021). Another study documented a 95% reduction in giant kelp canopy and a 2,485% increase in herbivore densities in a subregion of Baja California after extreme heatwaves, with shifts to urchin barrens as alternative stable states (Bauer et al., 2026). In Tasmania, early warning “incipient barrens” expanded along 500 km of coast, with projections that about 50% of kelp beds in the range extension region may collapse by ∼2030 (Ling and Keane, 2024). These changes are often abrupt, spatially contagious, and strongly linked to warming interacting with predator overfishing and herbivore release (Rogers-Bennett and Catton, 2019; Ling and Keane, 2024; McPherson et al., 2021; Bauer et al., 2026). These losses represent not only reductions in biomass but also irreversible changes in spatial organization, trophic interactions, and ecosystem complexity, which cannot be restored through simple redistribution of primary production.

Not all macroalgal responses are negative. Warming and eutrophication can also drive blooms and spatial expansion of some macroalgae: biomass of the brown alga *Sargassum horneri* in the East China and Yellow Seas increased from 2000 to 2021, primarily linked to ocean warming, while green alga *Ulva prolifera* increases appear to reflect combined warming and coastal human activities such as aquaculture (Qi et al., 2022). Tropical macroalgal reefs can be natural and productive components of tropical seascapes; high macroalgal cover is not always an anthropogenic symptom and may support fisheries and productivity where herbivore communities and connectivity remain intact (Fulton et al., 2019).

At the scale of whole marine and terrestrial assemblages, a global time series analysis reveals that rapid compositional change is “prevalent,” with marine biomes exceeding and terrestrial biomes trailing the overall trend, while richness change remains near zero on average in both realms (Blowes et al., 2019). This suggests that marine communities, including macroalgal systems, may be more dynamically reassembled under anthropogenic forcing, whereas terrestrial plant communities often show more clearly directional richness and abundance declines under intense, widespread land use change (Keck et al., 2025; Jaureguiberry et al., 2022; Newbold et al., 2015; Pärtel et al., 2025; Blowes et al., 2019).

### 3.3 Quantitative comparison: chronic, diffuse terrestrial pressures vs. episodic but intense marine macroalgal impacts

When comparing magnitude and mode of impact on terrestrial plants versus macroalgae, several quantitative contrasts emerge. First, spatial penetration and chronicity of human pressure are much higher on land. Around three-quarters of terrestrial area is measurably impacted (Venter et al., 2016), with a quarter of ecosystems increasing in modification over three decades (Theobald et al., 2025). Local plant communities in highly affected terrestrial regions retain less than one-fifth of their regionally suitable species (Pärtel et al., 2025), and severe land use change can cut local richness by ∼75% (Newbold et al., 2015). These patterns reflect close spatial proximity between human infrastructure, agriculture, and terrestrial vegetation, and emphasize land use change and exploitation as primary, continuous drivers (Jaureguiberry et al., 2022; Theobald et al., 2025; Newbold et al., 2015; Mu et al., 2022; Wilting et al., 2017; Venter et al., 2016; Pärtel et al., 2025).

By contrast, macroalgal systems experience more episodic, threshold type changes, often triggered by extreme climatic events and trophic cascades superimposed on more localized exploitation and pollution. Kelp forests can lose >90–95% of canopy area over hundreds of kilometers during multi-year marine heatwaves, followed by long lived urchin barrens with drastic reductions in primary productivity and associated biodiversity (Rogers-Bennett and Catton, 2019; McPherson et al., 2021; Bauer et al., 2026). Herbivore densities can increase by more than 20-fold in such transformed systems (Bauer et al., 2026), and abalone mortality can reach 80% (Rogers-Bennett and Catton, 2019). These sharp transitions contrast with the more gradual but pervasive loss of terrestrial plant richness and completeness across fragmented landscapes (Newbold et al., 2015; Pärtel et al., 2025).

Second, driver hierarchies diverge between realms. On land, statistical synthesis across indicators shows land use and direct exploitation as the top drivers, with climate change playing a relatively smaller yet growing role (Jaureguiberry et al., 2022; Newbold et al., 2015; Montràs-Janer et al., 2024). For marine foundation species (including kelps and other macroalgae), gradual warming and marine heatwaves already emerge as the most pervasive drivers, often acting interactively with overfishing and coastal development (Hanley et al., 2023; Rogers-Bennett and Catton, 2019; Ling and Keane, 2024; McPherson et al., 2021; Wernberg et al., 2023; Bauer et al., 2026). Marine macroalgae can also respond positively to warming via range expansions and blooms, as in *S. horneri* and *U. prolifera* (Qi et al., 2022), whereas terrestrial vascular plants more commonly respond to land use by net richness and abundance losses at local scales (Newbold et al., 2015; Pärtel et al., 2025).

Third, spatial structure of biodiversity change differs. Terrestrial plant communities show strong links between surrounding human footprint and local “dark diversity,” with higher regional human activity reducing the proportion of suitable species present locally even where vegetation appears relatively intact (Pärtel et al., 2025). Marine assemblages, in contrast, display stronger and more variable compositional changes, with marine biomes exceeding terrestrial ones in rates of turnover, yet without consistent net richness decline (Keck et al., 2025; Blowes et al., 2019). Macroalgal habitats can naturally form extensive, productive reefs near landmasses without heavy human modification, and high macroalgal dominance is not inherently symptomatic of degradation in all tropical systems (Fulton et al., 2019).

Finally, ecosystem service trajectories differ in how they intersect with exploitation. On land, human consumption—especially food—drives a large share (≈40%) of global biodiversity loss via land occupation and greenhouse gas emissions (Wilting et al., 2017). In the sea, macroalgae are both victims of stress and potential instruments for climate mitigation: restoration of wild seaweed forests, expansion of sustainable seaweed aquaculture, and use of seaweed products for emission abatement have been proposed as climate mitigation strategies, albeit with substantial uncertainties about net carbon sequestration and ecological side effects (Ross et al., 2023). This dual role contrasts with most terrestrial plant exploitation, where extensive land conversion and biomass removal are primarily drivers of biodiversity loss rather than tools for mitigation (Jaureguiberry et al., 2022; Theobald et al., 2025; Newbold et al., 2015; Wilting et al., 2017).

## 4 Atmospheric vs. oceanic CO_2_ increase: do surface oceans keep pace?

The research consistently shows a strong, though regionally variable, link between rising atmospheric CO_2_ and increases in surface ocean CO_2_ (expressed as partial pressure, pCO_2_). On multidecadal scales, most open and many coastal ocean regions show surface pCO_2_ trends similar to, or slightly lower than, atmospheric trends, implying sustained uptake of anthropogenic CO_2_.

### 4.1 Global and basin scale trends in atmospheric and surface ocean CO_2_

Long reconstructions of global open ocean pCO_2_ show increases of about +1.2 to +2.0 μatm yr^-1^ since ∼1990, with a mean global sea–air CO_2_ sink of about −1.76 PgC yr^-1^ for 1990–2012 and an increasing uptake over time (Iida et al., 2015; Takahashi et al., 2009). Over 1981–2010, most tropical and subtropical gyres display pCO_2_ trends parallel to or shallower than atmospheric CO_2_, consistent with long term equilibration to rising atmospheric CO_2_ (Fay and McKinley, 2013). Earth system model–data comparisons confirm that, for 1970–2011, surface ocean pCO_2_ growth rates are lower than atmospheric growth over ∼90% of the ocean, implying strengthening uptake; by 2100, models project that in >40% of the surface ocean pCO_2_ will rise faster than the atmosphere, weakening the sink (Tjiputra et al., 2014). Reviews of the global ocean sink estimate mean uptake of about −2.7 PgC yr^-1^ for 1990–2019, tripling since the 1960s and broadly matching atmospheric CO_2_ growth, but with substantial decadal variability driven by climate modes (Gruber et al., 2023; DeVries et al., 2023) (Table 3).

**TABLE 3:** Representative growth rates of atmospheric vs. surface ocean pCO_2_. Comparison of atmospheric and surface ocean CO_2_ trends in key regions.

Region/Metric	Atmospheric pCO_2_ trend (μatm yr^-1^)	Surface ocean pCO_2_ trend (μatm yr^-1^)	Interpretation	References
Global open ocean (1990–2012)	∼2 (implied)	+1.2 to +2.0 (regional)	Broadly keeps pace	Iida et al. (2015), Takahashi et al. (2009)
Gyre-scale tropics/subtropics (1981–2010)	∼1.5–2 (implied)	Parallel or shallower than air	Uptake maintained/increasing	Fay and McKinley (2013)
North pacific subtropical gyre (Hawaii, 2002–2019)	2.2 ± 0.1	1.7 ± 0.1	Ocean pCO_2_ rises slightly slower	Chen et al. (2021)
Bering sea basin (2003–2019)	2.1	2.8	Surface pCO_2_ outpaces air (reduced sink/increased source)	Zhang S. et al. (2023); Zhang M. et al. (2023)
South China sea (1992–2021)	Rising (not quantified)	1.19 ± 0.60	Largely atmosphere-driven increase	Zhang M. et al. (2023)
Southern California current offshore (2008–2022)	Rising (not quantified)	Trend “approximately follow [s]” atmosphere	Near air–sea equilibrium	Frazão et al. (2025)
Global coastal ocean (1982–2020)	Rising (not quantified)	Air–sea gradient increases	Coastal sink intensifying	Roobaert et al. (2024), Laruelle et al. (2018)

### 4.2 Coastal and shelf seas: often lagging the atmosphere

Analyses of continental shelves using SOCAT indicate that shelf pCO_2_ frequently lags atmospheric CO_2_, with the air–sea pCO_2_ gradient (ΔpCO_2_ = pCO_2_,air − pCO_2_,water) increasing in 653 of 825 grid cells, 325 significantly (Laruelle et al., 2018). This pattern implies an increasingly strong shelf CO_2_ sink as atmospheric CO_2_ rises faster than shelf waters equilibrate (Laruelle et al., 2018). An updated global coastal pCO_2_ product (1982–2020) finds the coastal ocean has acted as an average sink of −0.40 PgC yr^-1^, with sink strength increasing by 0.06 PgC yr^-1^ per decade; changes in the air–sea pCO_2_ gradient are a key driver of this trend (Roobaert et al., 2024). A broader synthesis estimates a global coastal sink of about −0.25 PgC yr^-1^, dominated (>90%) by polar and subpolar margins (Dai et al., 2022).

In specific shelf systems, trends reflect a mix of atmospheric forcing and regional processes. In the Bering Sea (2003–2019), atmospheric pCO_2_ increased by 2.1 μatm yr^-1^, while basin surface pCO_2_ rose faster (2.8 μatm yr^-1^), increasing emissions from the basin but with a strengthening sink on the continental shelf; overall, the integrated Bering Sea carbon sink increased from ∼6 to 19 TgC over 17 years (Zhang S. et al., 2023; Zhang M. et al., 2023). In the South China Sea, modeled surface pCO_2_ increased by ∼1.19 μatm yr^-1^, primarily linked to rising atmospheric CO_2_, with temperature dominating seasonal variability (Zhang M. et al., 2023). A comprehensive review concludes that coastal systems are, on average, a modest but increasing sink, with large spatial and temporal variability (Dai et al., 2022; Roobaert et al., 2024; Laruelle et al., 2018).

### 4.3 Open-ocean time series and regional case studies

At the Hawaii Ocean Time series in the North Pacific subtropical gyre, from 2002 to 2019, atmospheric pCO_2_ rose at 2.2 ± 0.1 μatm yr^-1^ and surface seawater pCO_2_ at 1.7 ± 0.1 μatm yr^-1^, indicating that the ocean surface there increased more slowly than the atmosphere and continued to absorb anthropogenic CO_2_ (Chen et al., 2021). Decomposition shows that the anthropogenic (atmospheric forced) component of surface pCO_2_ increased at 1.4 ± 0.1 μatm yr^-1^, partially offset by natural variability linked to large scale climate modes (Chen et al., 2021).

In the Southern California Current System, 2008–2022 moored records reveal that long term trends in surface pCO_2_ approximately track atmospheric CO_2_ at an offshore site, with dissolved inorganic carbon trends consistent with air–sea fluxes that maintain near equilibrium (Frazão et al., 2025). Nearshore, similar magnitude dissolved inorganic carbon trends may also reflect changing water mass composition and upwelling, complicating the simple air–sea equilibration picture (Frazão et al., 2025).

In the mid latitude North Atlantic (Porcupine Abyssal Plain), 2002–2016 data show no statistically significant long-term increase in seawater pCO_2_ despite rising atmospheric CO_2_; instead, the air–sea pCO_2_ difference increased, and the CO_2_ flux into the ocean strengthened by 0.19 ± 0.03 mmol m^-2^ day^-1^ per year, partly due to biological drawdown and changing buffer capacity (Macovei et al., 2020).

Southern Ocean studies emphasize both long term acidification and climate driven variability. A time series south of the polar front (1985–2021) shows subsurface anthropogenic CO_2_ accumulation and a transition of the region from a net annual CO_2_ source (∼+0.8 molC m^-2^ yr^-1^ in 1985) to a sink (−0.5 molC m^-2^ yr^-1^ in 2020), with winter surface fCO_2_ rising at rates close to or slightly below atmospheric trends (Metzl et al., 2024). Broader analyses find that Southern Ocean sink strength stagnated in the 1990s but reinvigorated after ∼2000, strongly influencing global sink variability (Gruber et al., 2023; Fay and McKinley, 2013; Landschützer et al., 2018; Yun et al., 2022). Observation based products show a systematic increase in winter–summer pCO_2_ differences of ∼2.2 μatm per decade poleward of 10°, driven mainly by anthropogenic CO_2_ uptake and modified by reduced buffer capacity (ocean acidification) (Landschützer et al., 2018), which amplifies seasonal extremes without changing the long-term tendency for uptake where surface pCO_2_ grows more slowly than the atmosphere.

### 4.4 Global ocean sink and the “keeping pace” question

Multiple global syntheses confirm that, over the past several decades, the ocean as a whole has absorbed about 25% of anthropogenic emissions, with mean contemporary sinks of ∼ −1.6 to −2.7 PgC yr^-1^ depending on period and method (DeVries et al., 2023; Gruber et al., 2023; Takahashi et al., 2009; Watson et al., 2020). Time resolved flux estimates from pCO_2_ products, corrected for near surface temperature gradients and cool skin effects, indicate stronger net uptake (by 0.8–0.9 PgC yr^-1^) than previously thought, bringing flux estimates into better agreement with observed increases in ocean carbon inventory (Watson et al., 2020).

From 2001 to 2018, observation based pCO_2_ products diagnose an accelerating sink of −0.61 ± 0.12 PgC yr^-1^ decade^−1^, while models attribute −0.34 to −0.41 PgC yr^-1^ decade^−1^ to rising atmospheric CO_2_ alone, implying an additional climate forced enhancement (DeVries et al., 2023). Inverse modeling suggests that rising atmospheric CO_2_ and associated physical controls (temperature, gas transfer) can explain the long-term net increase in ocean carbon (about 185 PgC from 1780 to 2020) but not the full decadal variability, which must involve circulation and biological changes redistributing carbon within the ocean (DeVries, 2022).

Surface ocean pH and buffer capacity reconstructions from 1750 to 2100 show that air–sea CO_2_ disequilibrium is the dominant spatial driver of surface pH patterns; areas where surface pCO_2_ lags the atmosphere exhibit stronger ongoing uptake and more rapid acidification (Jiang et al., 2019). Over recent decades, in many gyre scale biomes and coastal regions, surface pCO_2_ trends remain slightly below atmospheric trends, indicating that surface ocean CO_2_ is increasing, but in large parts of the ocean still not fully “catching up” with the atmosphere, thereby maintaining a net sink (Fay and McKinley, 2013; Iida et al., 2015; Laruelle et al., 2018; Roobaert et al., 2024; Chen et al., 2021; DeVries et al., 2023; Gruber et al., 2023).

## 5 Habitable space on land vs. in the ocean: how close are we to “saturation”?

Human use of Earth’s surface is rapidly expanding, but land and ocean are being “filled up” in very different ways. Terrestrial systems show deep, long-term transformation of habitable land, while marine systems are increasingly subject to overlapping human pressures rather than dense physical occupation. Comparing them requires looking at both physical space and planetary boundaries for safe use.

### 5.1 Terrestrial habitable land: extent, transformation, and limits

Over more than 10,000 years, land use has transformed most terrestrial ecosystems, shrinking wilderness, reshaping biodiversity and climate, and making the planet “largely transformed by land use” (Ellis, 2021). Recent mapping shows that by 2015, about 14.6% of global land area was directly and strongly modified and 1.6 million km^2^ of natural land had been lost since 1990, with rates of loss accelerating (Theobald et al., 2020). Human footprint analyses indicate that measurable human pressures now affect about 75% of Earth’s land surface (Venter et al., 2016), and more than two million km^2^ of terrestrial wilderness (Human Footprint <1) disappeared between 2000 and 2018, with mangrove biomes particularly affected (Mu et al., 2022).

Global land is increasingly scarce, as expanding urbanization and agriculture collide with limited arable land and regional resource constraints (Wang, 2022; Creutzig et al., 2019). Land system change is explicitly recognized as a planetary boundary: early work proposed keeping cropland below 15% of ice-free land (Rockström et al., 2009), and updated assessments find that the land system boundary, together with several other boundaries, is now transgressed (Richardson et al., 2023; Rockström et al., 2024). Feeding 9–10 billion people within these limits is possible only under stringent conditions: near vegan diets, fossil free energy, and no further cropland expansion (Schlesier et al., 2024; Schupp et al., 2019). Affluence also displaces land use to other countries via trade, so high income nations appropriate disproportionate biologically productive land and ocean area through imports (Weinzettel et al., 2013).

Habitability itself is historically understood as limited and uneven, with many regions now projected to become uninhabitable under climate change, reinforcing that truly habitable terrestrial space is more restricted than raw land area suggests (Fleetwood, 2023).

### 5.2 Terrestrial pressures vs. safe operating space

The planetary boundaries framework shows that multiple Earth system processes tied closely to land (biosphere integrity, biogeochemical flows, land system change, climate) are beyond the safe operating space, implying that the functional “habitable” capacity of land for stable human societies is already overdrawn (Rockström et al., 2009; Richardson et al., 2023; Rockström et al., 2024; Biermann and Kim, 2020).

This transgression reflects not only the magnitude of anthropogenic pressure but also the progressive loss of systemic buffering capacity within terrestrial ecosystems. As critical thresholds are exceeded, the ability of land systems to absorb disturbances without undergoing structural or functional reorganization becomes increasingly constrained. In practical terms, this translates into reduced resilience, diminished redundancy in ecological functions, and a higher probability of nonlinear responses to additional stressors.

Importantly, the notion of “safe operating space” is not solely a biophysical limit but also an emergent property of tightly coupled socio-ecological systems. Land-use intensification, fragmentation, and homogenization reduce the spatial heterogeneity that underpins ecosystem stability, thereby narrowing the margin within which human activities can expand without triggering destabilizing feedbacks. Even where physical land area remains available, its effective ecological functionality may already be compromised, reinforcing the distinction between geometric space and functionally viable space.

Furthermore, the convergence of multiple boundary transgressions amplifies systemic risk through cross-domain interactions. For example, alterations in biogeochemical cycles can exacerbate climate feedbacks, while biodiversity loss can weaken ecosystem regulation of carbon, water, and nutrient fluxes. These interactions suggest that terrestrial saturation is not a single-process phenomenon but a cumulative outcome of interacting constraints that collectively limit the capacity of land systems to sustain additional productive or regulatory functions.

Within this context, the concept of functional saturation gains particular relevance: terrestrial environments may reach a point at which incremental increases in use or productivity no longer translate into proportional gains, but instead accelerate degradation and instability. This condition implies that future expansions of terrestrial production or ecosystem services will increasingly depend on efficiency gains, restoration, or redistribution, rather than on the availability of new, unconstrained land space.

### 5.3 Marine and subaquatic space: use, impact, and “saturation”

The ocean covers the majority of Earth’s surface, and physically, direct human occupation of subaquatic space (e.g., permanent underwater settlements) is negligible. However, marine space is heavily used through fishing, shipping, energy, extraction, and coastal development. Europe’s seas illustrate the pattern: combined effects from multiple human pressures affect 96% of European marine area, with physical disturbance impacting 86% of coastal and 46% of shelf areas (Korpinen et al., 2021). Globally, cumulative impacts on the ocean are increasing: 59% of the ocean experienced significantly rising cumulative impact between 2003 and 2013, driven mainly by climate change but also by fishing, land-based pollution, and shipping, with coral reefs, seagrasses, and mangroves at greatest risk (Halpern et al., 2019).

Marine spatial planning has emerged to manage these intensifying, overlapping uses, and the concept of multi-use (co locating activities such as wind farms, aquaculture, tourism) seeks to optimize limited sea space while minimizing conflict (Schupp et al., 2019; Stancheva et al., 2022). These approaches acknowledge that, although three dimensional and vast, usable marine space near coasts and on productive shelves is becoming crowded in functional terms, even if not “settled” like land.

From a planetary-boundaries perspective, ocean linked limits (ocean acidification, biosphere integrity, nutrient cycles) are also under pressure. Ocean acidification is close to breaching its boundary, and updated analyses classify six of nine planetary boundaries as transgressed, including biosphere integrity that encompasses marine life (Richardson et al., 2023; Rockström et al., 2024). Nonetheless, physical saturation of marine habitable volume for human residence remains far away; the constraint is instead ecological integrity and cumulative stress rather than lack of geometric space.

A critical distinction must be made between the geometric extent of the ocean and the ecologically functional space available for primary production. While the ocean occupies the majority of Earth’s surface, only a relatively small fraction provides suitable conditions for sustained macroalgal growth (Gattuso et al., 2006; Duarte et al., 2022).

Macroalgal communities are largely restricted to shallow, light-penetrated coastal and shelf environments, where sufficient irradiance, appropriate substrate, and nutrient availability coexist (Duarte et al., 2022; Gattuso et al., 2006). In contrast, vast oceanic regions - including deep ocean basins, oligotrophic gyres, and sediment-dominated margins - are functionally unsuitable for macroalgal colonization due to light limitation, low nutrient concentrations, unstable substrates, or excessive depth (Gattuso et al., 2006; Markager and Sand-Jensen, 1992).

Consequently, the apparent spatial “openness” of the ocean is largely a geometric property rather than an indicator of biological production capacity. When evaluated in terms of ecologically viable habitat, marine macroalgal expansion is constrained to a comparatively narrow and already heavily utilized portion of the global ocean (Duarte et al., 2022; Gattuso et al., 2006).

### 5.4 Different saturation dynamics: functional vs. physical

We use the term functional saturation to describe a condition in which ecosystems, although not necessarily fully occupied in a geometric sense, are no longer able to support additional ecological functions or productivity without significant degradation, due to accumulated anthropogenic pressures and systemic constraints.

On land, humans occupy, farm, and build over a finite two-dimensional surface, with most ecologically productive and climatically favorable areas already heavily modified or under measurable pressure (Ellis, 2021; Mu et al., 2022; Venter et al., 2016; Theobald et al., 2020; Creutzig et al., 2019). This means that both physical and ecological “room” for expansion of conventional settlements is limited.

In the ocean, human activities spread thinly but widely, creating a near global footprint of stress without dense physical settlement (Korpinen et al., 2021; Halpern et al., 2019). Coastal and shelf zones, which are most relevant for human use and marine biodiversity, are approaching a functional saturation of allowable impact if planetary boundaries are to be respected, yet large volumes of deep ocean remain largely uninhabited and physically unoccupied.

### 5.5 Comparative view: which domain appears closer to exhaustion?

Across these dimensions, habitable terrestrial land is closer to physical and ecological saturation for conventional human settlement: most suitable land is already transformed or under active pressure, and expanding further risks deepening transgression of planetary boundaries (Ellis, 2021; Mu et al., 2022; Rockström et al., 2009; Venter et al., 2016; Richardson et al., 2023; Rockström et al., 2024; Theobald et al., 2020; Creutzig et al., 2019). Marine space, by contrast, is far from physically saturated as living space but is rapidly approaching ecological limits in many coastal and shelf areas due to rising cumulative impacts (Korpinen et al., 2021; Halpern et al., 2019; Richardson et al., 2023; Rockström et al., 2024) (Table 4).

**TABLE 4:** Key dimensions of “exhaustion” for land vs. ocean: comparison of land and ocean use saturation indicators.

Dimension	Terrestrial habitable land	Marine/subaquatic space	References
Direct human modification/footprint	∼75% of land under measurable human pressure; >14% strongly modified; wilderness rapidly declining	96% of european seas under combined effects; 59% of global ocean with increasing cumulative impact	Mu et al. (2022), Venter et al. (2016), Theobald et al. (2020), Korpinen et al. (2021), Halpern et al. (2019)
Planetary boundaries directly exceeded	Land-system change and biosphere integrity transgressed; multiple land-linked boundaries beyond safe space	Ocean acidification close to boundary; marine biosphere part of transgressed biosphere-integrity boundary	Rockström et al. (2009), Richardson et al. (2023), Rockström et al. (2024)
Resource scarcity for basic needs	Arable land and regional carrying capacity major constraints for feeding 9–10 billion sustainably	Food, energy, and transport uses increasing, but physical space for human habitation largely unused	Schlesier et al. (2024), Schupp et al. (2019), Wang (2022), Creutzig et al. (2019), Weinzettel et al. (2013), Korpinen et al. (2021), Halpern et al. (2019)
Trend in pressure	Long-term transformation; accelerating recent loss of natural lands and wilderness	Rapidly rising cumulative impacts, especially from climate and fishing; strong pressure on coastal ecosystems	Ellis (2021), Mu et al. (2022), Venter et al. (2016), Theobald et al. (2020), Halpern et al. (2019), Korpinen et al. (2021)

## 6 Discussion

The present synthesis brings into focus a fundamental question in Earth system ecology: whether the progressive contraction of terrestrial plant communities under intensifying anthropogenic pressure may be functionally offset by a concomitant expansion of marine macroalgal biomass in a high-CO_2_ world. Addressing this question requires integrating three interacting dimensions—anthropogenic spatial pressure, atmospheric–oceanic carbon dynamics, and the finite nature of habitable space—while accounting for the distinct ecological and biogeochemical constraints that govern terrestrial and marine primary producers.

A first-order observation emerging from the literature is that terrestrial and marine systems are undergoing profoundly different modes of anthropogenic influence (Crisp et al., 2022). Terrestrial environments are characterized by direct, structural transformation, in which land-use change physically displaces or fragments plant communities. The extent of this transformation is now planetary in scale, with approximately three-quarters of the land surface experiencing measurable human pressure and a substantial fraction already strongly modified. This process constitutes not merely a redistribution of vegetation but a contraction of viable ecological space, often accompanied by declines in biodiversity, simplification of community structure, and disruption of long-established phytosociological assemblages (Petrescu-Mag, 2026). The concept of “habitable land” must therefore be interpreted not only in geometric terms but also through the lens of ecological functionality, which is increasingly constrained as planetary boundaries related to land-system change and biosphere integrity are exceeded. Such constraints are consistent with long-term Earth-system analyses showing that environmental variables and Earth-life interactions exhibit characteristic statistical structures indicating that planetary habitability is regulated by persistent dynamical constraints rather than by instantaneous environmental conditions alone (Shang, 2024a; Shang, 2024b).

In contrast, marine environments, while heavily impacted, are not subject to the same degree of direct spatial occupation (Wang et al., 2022). Human activities in the ocean are extensive but predominantly diffuse, encompassing fishing, shipping, nutrient loading, and climate-driven stressors rather than wholesale physical replacement of ecosystems (Petrescu-Mag et al., 2023). Even in regions where cumulative impacts are high, the geometric availability of space remains vast, and macroalgal communities are rarely excluded in the same absolute sense as terrestrial vegetation. This distinction creates a theoretical asymmetry: while terrestrial systems are approaching both physical and functional saturation, marine systems retain spatially heterogeneous and environmentally constrained opportunities for biological expansion, primarily within coastal and shelf environments. The apparent spatial openness of marine systems should not be interpreted as an absence of ecological limitation, but rather as a difference in the type and distribution of constraints compared to terrestrial systems.

Within this asymmetrical framework, rising atmospheric CO_2_ emerges as a pivotal driver with dual and partially opposing effects on marine primary producers. Increased CO_2_ availability can alleviate carbon limitation in photosynthesis, particularly for macroalgae lacking highly efficient carbon concentrating mechanisms, thereby enhancing growth rates and biomass accumulation. At the same time, the dissolution of CO_2_ into seawater drives ocean acidification, altering carbonate chemistry, reducing pH, and imposing physiological stress on a wide range of marine organisms (Ma et al., 2023). The interaction between these processes is governed by air–sea exchange dynamics, which introduce an important temporal dimension into the system.

From a mechanistic perspective, the response of marine primary producers to rising atmospheric CO_2_ is governed by changes in the seawater carbonate system rather than by a simple increase in dissolved CO_2_ availability (Hurd et al., 2020; García-Ibáñez et al., 2022). The uptake of atmospheric CO_2_ alters the distribution of dissolved inorganic carbon species (CO_2_(aq), HCO_3_^−^, CO_3_^2-^), reduces seawater pH, and decreases carbonate saturation states, thereby modifying both carbon acquisition pathways and calcification processes (Hurd et al., 2020; Kawahata et al., 2019; Petton et al., 2024; Zhang Z. et al., 2024).

Importantly, macroalgae are not functionally uniform in their responses to these changes (Hepburn et al., 2011; Hurd et al., 2020; Porzio et al., 2011). Many brown algae (e.g., kelps) and some green algae possess efficient carbon-concentrating mechanisms (CCMs) that allow the utilization of bicarbonate, potentially reducing their sensitivity to increased CO_2_ availability (Hepburn et al., 2011; Capó-Bauçà et al., 2024; Sun et al., 2023; Zhang X. et al., 2024). In contrast, species lacking strong CCMs may experience enhanced photosynthetic rates under elevated CO_2_ conditions (Hepburn et al., 2011; Raven, 2022).

Conversely, calcifying macroalgae, such as coralline red algae, are particularly vulnerable to declining carbonate saturation states, which can impair calcification and structural integrity (Büdenbender et al., 2011; Cornwall et al., 2021; Raven, 2022). These contrasting physiological responses imply that future increases in CO_2_ are likely to drive shifts in macroalgal community composition rather than a uniform increase in biomass (Hepburn et al., 2011; Porzio et al., 2011; Noonan et al., 2025).

Empirical observations indicate that surface ocean pCO_2_ generally increases in close correspondence with atmospheric trends, but often with a slight lag. This lag reflects the ocean’s buffering capacity and the kinetics of gas exchange, and it has two significant ecological implications (Jiang et al., 2024). First, it creates a transient phase during which macroalgal communities may experience elevated CO_2_ concentrations without the full expression of acidification stress, potentially enhancing productivity. Second, as atmospheric CO_2_ continues to rise and ocean buffering capacity diminishes, this lag narrows, leading to progressively stronger acidification effects that may counterbalance or even override the initial fertilization benefits. The result is not a stable enhancement of marine productivity, but rather a temporally dynamic response characterized by shifting trade-offs between resource availability and physiological stress.

The question of whether increased macroalgal productivity can compensate for terrestrial vegetation loss must therefore be evaluated not only in terms of gross primary production, but also in terms of community composition, physiological heterogeneity, and biogeochemical functionality. Marine macroalgal systems, particularly kelp forests and other foundation species assemblages (Adrias et al., 2025; Soumantri et al., 2024; Kepel et al., 2024), are increasingly recognized as structurally dynamic and susceptible to abrupt regime shifts. Diseases such as ice-ice can cause major biomass losses in cultivated macroalgae (Rahman et al., 2023; Rahman et al., 2025). Climate-driven disturbances such as marine heatwaves, in combination with trophic imbalances and local stressors, can trigger transitions to alternative stable states, including low-productivity barren systems (Sujatmiko et al., 2025). These transitions are often persistent and can significantly reduce both biomass and ecological function, undermining the reliability of macroalgae as compensatory primary producers.

Moreover, the biogeochemical role of macroalgae differs fundamentally from that of terrestrial plants. While macroalgal communities can exhibit high rates of primary production, a substantial proportion of this biomass is rapidly remineralized within the marine environment. Only a fraction is exported to deep ocean sinks where long-term sequestration can occur (Jiang et al., 2024; Petrescu-Mag et al., 2023). Consequently, the capacity of macroalgae to offset atmospheric CO_2_ increases is constrained by ocean circulation, decomposition processes, and ecological feedbacks, limiting their effectiveness as a global carbon sink despite high local productivity (Petrescu-Mag et al., 2023). This distinction is critical, as compensation for terrestrial loss would require not only equivalent production but also comparable contributions to long-term carbon storage and ecosystem stability.

It is important to distinguish between natural macroalgal ecosystems and industrial seaweed cultivation systems when discussing blue carbon dynamics. Natural macroalgal communities (e.g., kelp forests) contribute to carbon cycling through *in situ* production, partial export, and long-term sequestration pathways that are still not fully quantified. In contrast, industrial seaweed cultivation involves managed biomass production, harvesting, and often rapid carbon re-release depending on end use, which may limit long-term sequestration (Nuryadi et al., 2024; Kamlasi et al., 2025). Moreover, ecological consequences differ substantially, as natural systems support complex habitats and biodiversity, whereas large-scale cultivation may introduce localized ecological pressures and biogeochemical alterations.

An additional layer of complexity arises from the interaction of multiple stressors in marine systems (Yoshida et al., 2025; Patadjai et al., 2024). Ocean warming, deoxygenation, nutrient enrichment, and acidification do not operate in isolation but interact in ways that can amplify their individual effects. These interactions often produce nonlinear and region-specific responses, further complicating predictions of macroalgal expansion. In many cases, the net effect of these stressors is negative, particularly for habitat-forming species that underpin complex marine ecosystems. Thus, while opportunistic or bloom-forming algae may increase under certain conditions, these do not necessarily provide equivalent ecological functions to the structured plant communities being lost on land.

The temporal dimension of these processes also reveals a critical mismatch between terrestrial degradation and marine response. Land-use change operates on relatively rapid timescales, leading to immediate and often irreversible loss of plant communities. In contrast, marine responses to elevated CO_2_ and associated environmental changes unfold more gradually due to the lag in oceanic carbon uptake. This creates a period of systemic disequilibrium in which terrestrial primary production is declining or becoming spatially constrained, while marine productivity may be temporarily enhanced but remains unstable and subject to increasing stress. Such a mismatch precludes a synchronous or balanced compensation between the two domains.

This temporal asymmetry is further reinforced by the contrasting mechanisms governing long-term carbon storage in marine systems (Nowicki et al., 2022; Ricour et al., 2023; Santos et al., 2021; Meilijson et al., 2023). While terrestrial ecosystems can accumulate carbon in biomass and soils over decadal to centennial timescales (Von Fromm et al., 2025; Qiu et al., 2023; Spohn, 2020; Xu et al., 2019), effective long-term sequestration in the ocean depends on processes such as particulate organic carbon export, deep ocean circulation, and sediment burial (Shang, 2023; Hansell et al., 2012; Bradley et al., 2022; Meilijson et al., 2023). These processes can support carbon storage over much longer timescales, often ranging from centuries to millennia (Shang, 2023; Nowicki et al., 2022; Filbee-Dexter et al., 2024; Santos et al., 2021; Bradley et al., 2022; Piñeiro-Juncal et al., 2025).

As a result, even if marine primary production were to increase in the short term, the translation of this production into stable, long-term carbon storage is delayed and highly uncertain. This decoupling between production and sequestration further limits the capacity of marine systems to compensate for rapid terrestrial losses.

Furthermore, the apparent abundance of marine space must be interpreted cautiously. Although the ocean is not physically saturated, the regions most conducive to high macroalgal productivity—namely coastal and shelf areas—are precisely those experiencing the greatest cumulative human impacts (Adrias et al., 2025). These zones are approaching functional limits in terms of ecological integrity, even if they are not geometrically occupied. As a result, the effective habitat available for sustained macroalgal expansion is more limited than global ocean area alone would suggest.

Taken together, these considerations indicate that the potential for marine macroalgae to compensate for the retreat of terrestrial plant communities is inherently constrained. While localized increases in macroalgal biomass and productivity are plausible under elevated CO_2_ conditions, these increases are unlikely to match the scale, structural complexity, and ecological stability of terrestrial vegetation losses. The differences in spatial organization, biogeochemical function, and susceptibility to disturbance between the two systems preclude a simple substitution.

The broader implication is that the Earth system is not undergoing a balanced redistribution of primary producers from land to sea, but rather a transition toward a new configuration characterized by reduced terrestrial complexity and increasingly stressed marine systems. The rise in atmospheric CO_2_ may transiently enhance certain aspects of marine productivity, but this effect is tightly coupled to processes that ultimately constrain and destabilize marine ecosystems. In this context, the decline of terrestrial phytosociological systems represents not a shift in location of primary production, but a net reduction in the resilience and integrity of the biosphere as a whole. A summary of the discussion is presented in Figure 1.

**FIGURE 1:**
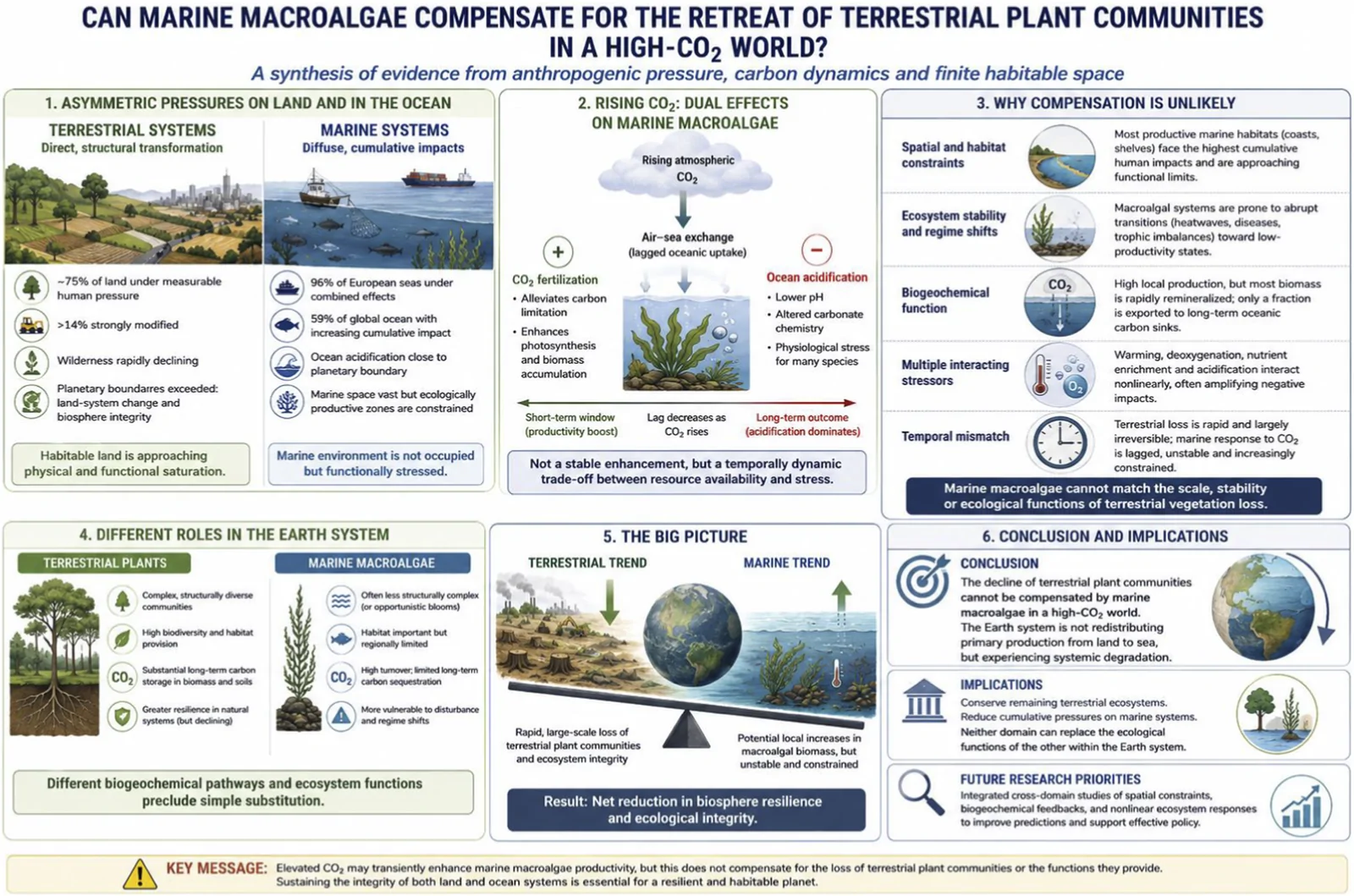
Can macroalgae compensate for the retreat of terrestrial plant communities in a high-CO_2_ world? (original plate, designed by I. V. Petrescu-Mag, edited using OpenAI).

## 7 Conclusion

The synthesis developed in this review leads to a clear and structurally consistent conclusion: the decline of terrestrial plant communities driven by anthropogenic land-use change cannot be functionally or quantitatively compensated by the expansion of marine macroalgal systems, even under conditions of elevated atmospheric CO_2_.

At the core of this conclusion lies a fundamental asymmetry between terrestrial and marine domains. Terrestrial ecosystems are approaching both physical and ecological saturation, with most habitable land already transformed or subjected to significant human pressure. This process results not only in spatial limitation but also in a profound erosion of ecological functionality, biodiversity, and structural complexity. In contrast, while the marine environment remains geometrically extensive, its ecologically productive zones—particularly coastal and shelf regions—are increasingly constrained by cumulative anthropogenic impacts, limiting their effective capacity to support sustained macroalgal expansion.

The role of rising atmospheric CO_2_ further reinforces this asymmetry rather than resolving it (Halloran et al., 2025). Although increased CO_2_ availability can transiently stimulate macroalgal photosynthesis, this effect is inherently unstable and counterbalanced by ocean acidification, warming, deoxygenation, and other interacting stressors. The temporal lag in oceanic CO_2_ equilibration introduces a short-lived window of enhanced productivity, but this is progressively diminished as acidification intensifies and buffering capacity declines. Consequently, marine primary production does not exhibit a stable or scalable trajectory capable of offsetting terrestrial losses.

Moreover, the biogeochemical functions of terrestrial vegetation and marine macroalgae are not interchangeable. Terrestrial plants contribute significantly to long-term carbon storage through biomass accumulation and soil formation, whereas a substantial fraction of macroalgal production is rapidly remineralized, with only limited transfer to long-term oceanic carbon sinks. This disparity critically limits the capacity of marine systems to compensate for terrestrial carbon sequestration deficits.

The susceptibility of macroalgal systems to abrupt regime shifts introduces an additional layer of uncertainty and instability. Climate-driven disturbances and trophic imbalances can rapidly transform productive ecosystems into low-functioning states, undermining both biomass accumulation and ecological services. Such nonlinear dynamics contrast with the already realized and often irreversible loss of terrestrial plant communities, creating a temporal and functional mismatch that precludes effective compensation.

Taken together, these findings indicate that the Earth system is not undergoing a redistribution of primary production from land to sea, but rather a systemic degradation characterized by reduced resilience, diminished ecological integrity, and increasing instability across both domains. The notion of marine macroalgae as a compensatory mechanism for terrestrial vegetation loss is therefore not supported under current understanding of Earth system constraints.

Future research should prioritize integrated, cross-domain analyses that explicitly account for spatial constraints, biogeochemical feedbacks, and nonlinear ecosystem responses. From a policy perspective, the results underscore the urgency of conserving remaining terrestrial ecosystems while simultaneously mitigating cumulative impacts on marine environments, recognizing that neither domain can substitute for the ecological functions of the other within the Earth system.
